# Oyster Peptides Ameliorate Dextran Sulfate Sodium-Induced Ulcerative Colitis via Modulating the Gut Microbiota and Inhibiting the TLR4/NF-κB Pathway

**DOI:** 10.3390/nu16111591

**Published:** 2024-05-23

**Authors:** Haixiang Guo, Wenyin Xie, Zhonghao Ji, Bingbing Wang, Wenzhi Ren, Wei Gao, Bao Yuan

**Affiliations:** 1 Department of Laboratory Animals, College of Animal Sciences, Jilin University, Changchun 130062, China; hxguo23@mails.jlu.edu.cn (H.G.); wyxie22@mails.jlu.edu.cn (W.X.); jizh21@mails.jlu.edu.cn (Z.J.); wangbb23@mails.jlu.edu.cn (B.W.); renwz@jlu.edu.cn (W.R.); 2 Department of Basic Medicine, Changzhi Medical College, Changzhi 046000, China

**Keywords:** ulcerative colitis, oyster peptide, intestinal barrier, intestinal microbiome

## Abstract

Ulcerative colitis (UC) is an inflammatory bowel disease with an increasing prevalence year over year, and the medications used to treat patients with UC clinically have severe side effects. Oyster peptides (OPs) have anti-inflammatory and antioxidant properties as functional foods that can alleviate a wide range of inflammatory conditions. However, the application of oyster peptides in ulcerative colitis is not well studied. In this work, an animal model of acute colitis was established using 3% dextran sulfate sodium (DSS), and the impact of OP therapy on colitis in mice was examined. Supplementing with OPs prevented DSS-induced colitis from worsening, reduced the expression of oxidative stress and inflammatory markers, and restored the intestinal barrier damage caused by DSS-induced colitis in mice. The 16S rDNA results showed that the OP treatment improved the gut microbiota structure of the UC mice, including increasing microbial diversity, increasing beneficial bacteria, and decreasing harmful bacteria. In the UC mice, the OP therapy decreased the relative abundance of Family_XIII_AD3011_group and Prevotella_9 and increased the relative abundance of Alistipes. In conclusion, OP treatment can inhibit the TLR4/NF-κB pathway and improve the intestinal microbiota in UC mice, which in turn alleviates DSS-induced colitis, providing a reference for the treatment of clinical UC patients.

## 1. Introduction

The common chronic inflammatory illness ulcerative colitis (UC) is characterized by weight loss, diarrhea, and rectal bleeding [1,2]. The frequency and incidence of UC have grown recently on a global scale, progressively turning the illness into a global health issue [3]. The pathogenesis of UC is complex and related to genetics, diet, immune system disorders, and gut microbiota disorders, but the exact pathogenesis of the disease remains unclear [4]. Current therapeutic agents for UC patients, such as corticosteroids, aminosalicylates, and immunosuppressants, frequently produce a wide variety of side effects in the clinic [5]. For example, corticosteroids may cause symptoms such as fever and rash in patients with UC [6]. Therefore, there is an urgent need to develop natural products that do not produce serious side effects as alternative therapies for UC treatment.

The main feature of UC is an impaired intestinal mucosal barrier, including impaired tight junctions, decreased mucus secretion, abnormal inflammatory cells, and an imbalance of intestinal microorganisms [7]. The first line of defense against dangerous chemicals is the intestinal epithelial barrier, which is made up of intestinal epithelial cells and tight junctions [8]. Impairment of intestinal barrier function, characterized by a decrease in tight junction proteins, is one of the pathogenic mechanisms of UC [9]. Moreover, an intestinal microbial community change is a risk factor for patients with UC due to disruption of the intestinal epithelial barrier [10]. It has been shown that the gut microbiota of patients with UC typically has a low abundance of beneficial bacteria and a high abundance of harmful bacteria, which usually leads to intestinal microecological dysbiosis and in turn promotes UC progression [11,12]. Maintaining the integrity of the intestinal barrier, promoting normal development of the mucosal immune system, and lowering pathogen invasion are all facilitated by a healthy gut microbiota [13]. It has been demonstrated that changing the gut microbiota’s composition is a successful way to treat UC [10]. DSS-induced UC mouse models exhibit similarities to the disease in humans and are among the most commonly used mouse models in UC [14,15]. Therefore, in order to examine the mechanism of action of OPs in reducing colitis in mice, we constructed an animal model of UC using DSS in this study.

Oysters are rich in vitamins, minerals and proteins and are a great source of high-quality nutrients found in many areas [16]. Oysters have high levels of health benefits as functional products. It has been demonstrated that polysaccharides, taurine, and peptides found in oyster extracts can reduce the symptoms of DSS-induced colitis in mice [17,18]. Among these, oyster polysaccharides modulate the gut microbiota to alleviate DSS-induced colitis and have strong anti-inflammatory activities [19]. Taurine is likewise effective in the alleviation of DSS-induced colitis in mice [20]. In addition, the results of several studies have emphasized the multiple physiological activities of oyster extract peptides, including antioxidant, immunomodulatory, anti-inflammatory, and anti-fatigue effects [21,22,23]. Studies on the function of oyster peptides and their regulatory pathways in the treatment of colitis are still scarce, nevertheless.

In this study, we identified small-molecule peptides in OPs that may play a potential role. An animal model of ulcerative colitis in mice was constructed using 3% DSS. By detecting the symptoms of colitis, the degree of intestinal barrier damage, and the changes of intestinal microbiota in mice, we were able to elucidate the mechanism of action of OPs in alleviating colitis in mice. The results of this study provide insights into the role of OPs in alleviating colitis in mice and provide a theoretical basis for the application of OPs in the prevention and treatment of colitis.

## 2. Materials and Methods

### 2.1. Materials and Reagents

DSS was obtained from MP Biomedicals (Irvine, CA, USA). The supplier of the claudin-1 antibody was Affinity (Affinity, Changzhou, China). GAPDH TLR4, p65, and *p*-p65 antibodies were obtained from Cell Signaling Technology (Cell Signaling Technology, Shanghai, China). OPs were provided by Tiantianhao Biological Products Co., Ltd. (Tiantianhao Biological Products, Wuhan, China) and were obtained by the enzymatic digestion of oysters.

### 2.2. Analysis of Amino Acid Composition

By adding hydrochloric acid to a final concentration of 6 mol/L, the BCA method was used to first determine the OP concentration. It was hydrolyzed under airtight conditions at 110 °C for 20–24 h, evaporated to dryness, and then reintroduced with hydrochloric acid to resolubilize and filter. After adding 10 μL of the hydrolyzed sample to the derivatization tube, 20 μL of the derivatizing agent and 70 μL of boric acid buffer were added one after the other. The sample was then heated for ten minutes at 55 °C in an oven before being utilized for liquid chromatography. The liquid chromatographic conditions were as follows: column temperature, 37 °C; flow rate, 1.0 mL/min; and UV wavelength, 248 nm. Finally, it was passed through an AccQ.

### 2.3. OP Peptide Identification

The OPs were first fully dissolved in 0.1% trifluoroacetic acid (TFA) to complete the desalting process. After dissolving the peptides with 20 μL of dissolution solution and centrifuging at 13,500 rpm for 20 min, the supernatant was moved to a sample tube so that it could be identified using mass spectrometry.

In the liquid chromatographic test, an 80% acetonitrile (CAN) and a 0.1% formic acid solution made up mobile phase B and mobile phase A, respectively. Gradients of 3–8% B over 7 min, 8–32% B over 39 min, 32–44% B over 5 min, and 44–99% B over 5 min were used to separate the samples. The primary mass spectrum had a resolution of 120,000 and a scanning range of 350–1550 m/z. The secondary mass spectral resolution was 30,000. Finally, PEAKS Studio 10 software was used for database searching.

### 2.4. Animals

Liaoning Changsheng Co. (Liaoning Changsheng, Shenyang, China) supplied male BALB/c mice that were 6 weeks old and in good health. The Institutional Animal Care and Use Committee of Jilin University (License No. SY202307004) authorized all experimental protocols. Regarding the choice of OP concentration, we referred to the concentration gradients used in other colitis studies [24], conducted a pilot experiment, and finally chose 500 mg/kg as the treatment concentration for our formal experiment.

Following a week of animal acclimation, the mice were split into three groups at random: (1) Con, the Control group, in which the mice were subjected to gavage with a 0.9% Nacl solution; (2) DSS, the DSS-induced colitis group, which was subjected to gavage with a 0.9% Nacl solution, and in the last week, the DSS was added to the drinking water (3%); (3) OP, the oyster-peptide-treated group, treated with OPs (500 mg/kg/d) by gavage, and DSS was added to drinking water (3%) in the last week. Throughout the previous week, the mice’s body weights were noted every day, and following their execution, blood and tissues were gathered.

### 2.5. Sample Collection, DAI Scoring, and Histopathology

A slice of the colon was removed for staining with H&E and AB-PAS, and two smaller sections were removed for Western blotting and RT-qPCR tests, respectively. Furthermore, the cecum’s contents were removed, quickly frozen in liquid nitrogen, and kept at −80 °C until additional testing could be conducted. Throughout the trial, the mice’s disease activity index (DAI) scores were collected and evaluated every day, and their overall health was checked. The DAI scores were determined by assessing the clinical signs of the mice according to the method described in previous studies [25].

The tissues from the collected mouse colon were sectioned after paraffin embedding, fixed in a 4% paraformaldehyde solution, and stained with hematoxylin and eosin (H&E). Alcian blue (AB) and periodic acid Schiff (PAS) staining were used to identify goblet cells. The pathological evaluation was performed according to previous methods [26], and the histological score was based on mucosal edema, crypt destruction and loss, and inflammatory cell infiltration. The number of goblet cells was measured using ImageJ software (version 1.6.0).

### 2.6. Enzyme-Linked Immunosorbent Assays (ELISAs)

An IL-6 ELISA kit (YX-E20012), a TNF-α ELISA kit (YX-E20220), an IL-1β ELISA kit (YX-E20533), an LPS ELISA kit (YX-121619M), an MDA assay kit (YX-E20347), a SOD assay kit (YX-E20348), and a T-AOC assay kit (YX-E21710) were obtained from Shanghai SINO-BESTBIO Co., Ltd. (Shanghai, China). Following the manufacturer’s instructions, the levels of cytokines (TNF-α, IL-6, and IL-1β) and oxidative stress factors (SOD, MDA, and T-AOC) were measured in serum and colon samples.

### 2.7. mRNA Expression Level Measurement

Using a Total RNA Extraction Kit (SM130, Sevenbio, Beijing, China), RNA was isolated from colon tissues. The MonScript^TM^ RTIII ALL-in-One Mix with dsDNase kit (Monad, Wuhan, China) was used to synthesize first-strand cDNA. A MonAmp^TM^ ChemoHS qPCR Mix (Monad, Wuhan, China) kit was used for RT-qPCR. Supplementary Table S1 contains all of the primer sequences needed for the RT-qPCR procedure.

### 2.8. Western Blotting

Using a BCA protein assay kit, the total protein content was ascertained after the total protein was isolated from colon tissue. Using the PAGE Gel Rapid Preparation Kit (Yase, Shanghai, China), proteins were isolated and then put onto PVDF (polyvinylidene difluoride) membranes (0.45 μm; Millipore, St. Louis, MO, USA). Specific binding was performed using specific antibody incubation, and sheep anti-rabbit secondary antibody was obtained from Yase (Shanghai, China). Finally, a quantitative analysis was performed using ImageJ software.

### 2.9. The 16S rDNA Gene Sequencing

After PCR amplification, DNA was extracted from the cecum contents using the CTAB technique and purified using AMPure XT beads (Beckman Coulter Genomics, Danvers, MA, USA). The PCR products were then assessed using Illumina (Kapa Biosciences, Woburn, MA, USA) library quantification kits and an Agilent 2100 Bioanalyzer (Agilent, CA, USA). For qualified libraries, optimized data were obtained through quality control, denoising, and splicing. Numerous studies, including species taxonomic, community diversity, and species difference analyses, were carried out using the ASV (feature) feature sequences and ASV (feature) abundance tables as a basis. For Alpha diversity, species richness and evenness were mainly reflected by Chao1, Observed OTU, Shannon, and Simpson indices [27]. For Beta diversity, differences between groups were mainly observed by Principal Component Analysis (PCA), Principal Coordinate Analysis (PCoA), and Non-Multidimensional Scale Analysis (NMDS) [28,29].

### 2.10. Network Diagram of Correlation Analysis

The correlation analysis methods used here refer to our previous studies [30,31,32]. Briefly, the correlations of DAI scores, body weight changes, differential bacteriophages, inflammation, and oxidative stress metrics in mice were analyzed and network plotted using OmicStudio tools (https://www.omicstudio.cn/tool, accessed on 16 October 2023).

### 2.11. Statistical Analysis

In this study, every experiment was conducted at least three times. Two sets of data were compared for significance using GraphPad Prism 9.5 *t* tests, and multiple comparisons of data significance were examined with a GraphPad Prism 9.5 one-way ANOVA. The means ± standard deviations of three separate biological replicates were used to represent all the data. The results were deemed statistically significant when *p* < 0.05.

## 3. Results

### 3.1. OPs Improve Colitis Symptoms in UC Mice

We determined the amino acid composition of OPs, with the highest levels being glycine, glutamic acid, and alanine. The detailed results can be found in Supplementary Table S2. The peptide composition results can be found in Supplementary Table S3.

**Figure 1:**
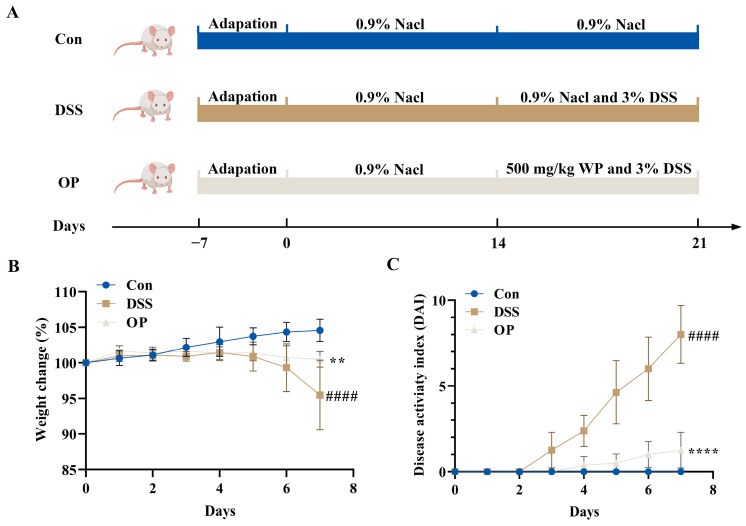
Effect of OP supplementation on DSS−induced colitis symptoms in mice. (**A**) Experimental groups, *n* = 8. (**B**) Daily change in body weight (%) of mice during DSS treatment, *n* = 8. (**C**) Daily change in DAI score of mice during DSS treatment, *n* = 8. **, *p* < 0.01. ****, *p* < 0.0001. ####, *p* < 0.0001.

An animal model of colitis was created in BALB/c mice using 3% DSS in order to examine the impact of OPs on the development of DSS-induced colitis (Figure 1A). The findings demonstrated that mice in the DSS and OP groups lost varying amounts of weight within 7 days of receiving DSS therapy (Figure 1B). The mice in the DSS group had DAI scores that were substantially greater than those in the control group, whereas the OP treatment significantly reduced the DAI (Figure 1C). Together, these results indicated that a 500 mg/kg OP treatment significantly suppressed the symptoms of colitis.

### 3.2. OPs Ameliorate Colonic Tissue Injury in UC Mice

**Figure 2:**
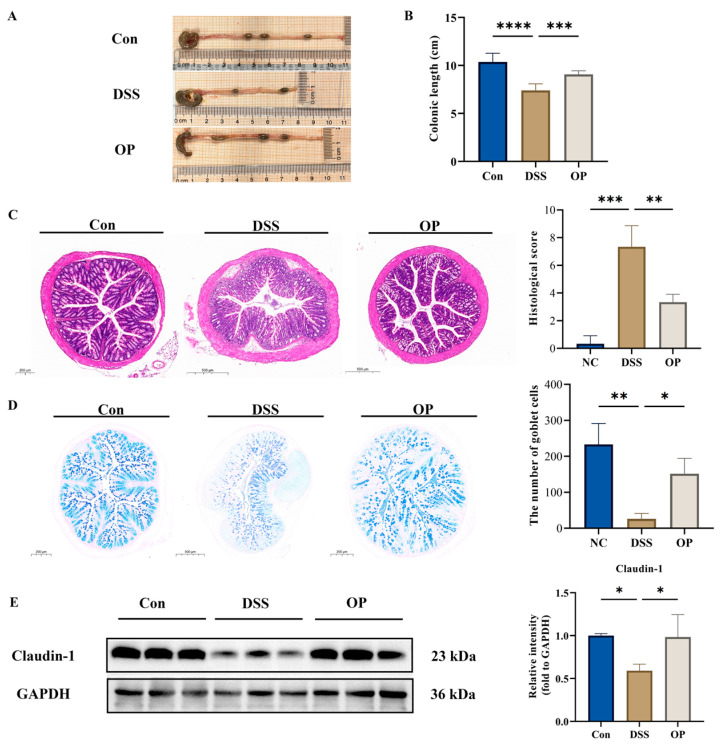
Effect of OP supplementation on tissue damage in UC mice. (**A**,**B**) Changes in colon length in the three groups of mice, *n* = 8. (**C**) H&E staining and histopathology scores in three groups of mice. (**D**) AB-PAS staining and number of goblet cells in three groups of mice. (**E**) Differential expression of the tight junction protein claudin-1, *n* = 3. *, *p* < 0.05. **, *p* < 0.01. ***, *p* < 0.001. ****, *p* < 0.0001.

Shortened colon length is an important indicator of the severity of colitis in UC mice [33]; the DSS treatment significantly shortened colon length and the OP treatment significantly ameliorated colon shortening due to DSS treatment (Figure 2A,B). A histologic examination of the intestine by H&E staining showed that the OP treatment restored the inflammatory cell infiltration and crypt developmental abnormalities caused by DSS (Figure 2C). Colon tissue slices were stained with AB-PAS to evaluate the impact of OPs on the intestinal barrier. The results showed that the OP treatment increased the number of goblet cells and restored the intestinal barrier (Figure 2D). We also looked at the tight junction protein claudin-1 expression simultaneously and found that it was substantially higher in the OP group than in the DSS group (Figure 2E).

### 3.3. OPs Inhibit Oxidative Stress Levels and Inflammatory Responses in UC Mice

**Figure 3:**
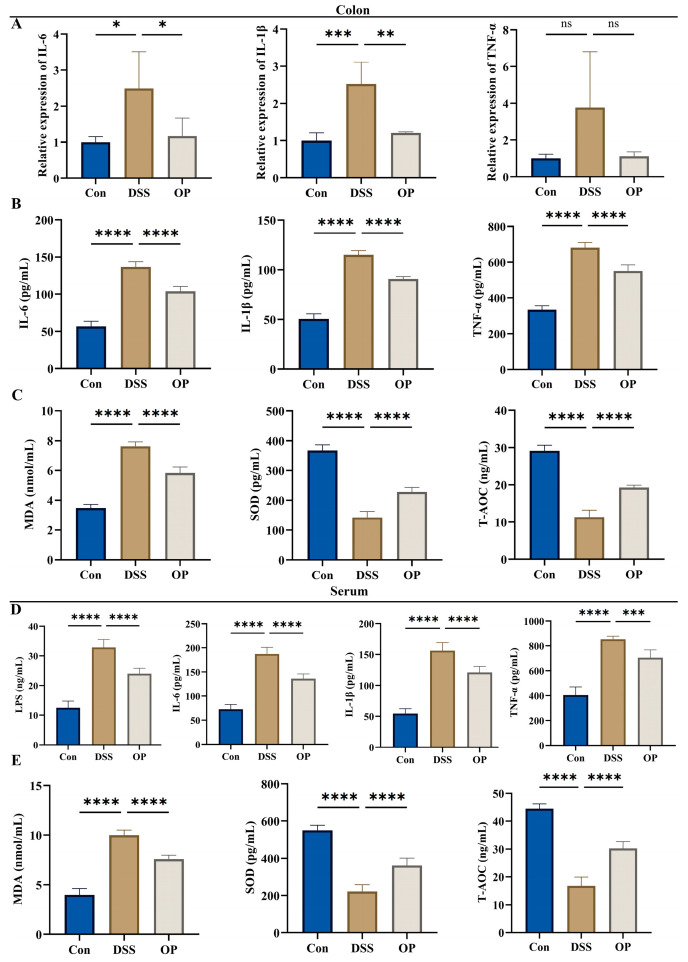
Effect of OP supplementation on the inflammatory response and oxidative stress in UC mice. (**A**) Quantitative results of three inflammatory factors in colonic tissues, *n* = 6. (**B**,**C**) Expression of colonic IL-6, IL-1β, and TNF-α and serum LPS, IL-6, IL-1β, and TNF-α using ELISA kits, *n* = 6. (**D**,**E**) Expression of colonic MDA, SOD, and T-AOC and serum MDA, SOD, and T-AOC markers, *n* = 6. ns, *p* > 0.05. *, *p* < 0.05. **, *p* < 0.001. ***, *p* < 0.0001. ****, *p* < 0.0001.

When we looked at the mRNA levels of inflammatory markers in colonic tissues, we discovered that OP therapy reduced their expression (Figure 3A). Consistent with the quantitative findings, ELISA was next used to detect the amounts of IL-6, IL-1β, and TNF-α in the colonic tissues of the three groups of mice (Figure 3B). In addition to the three inflammatory factors in serum, we also examined the levels of LPS and found that the OP treatment significantly suppressed LPS levels in the UC mice (Figure 3C). Furthermore, ELISA was used to measure alterations in MDA, SOD, and T-AOC expression in the colon and serum of three different mouse groups in order to evaluate the impact of OP on oxidative stress in colitis-affected animals. In the colon and serum of the OP group, MDA expression was much lower than in the DSS group, but SOD and T-AOC expression was significantly increased (Figure 3D,E).

### 3.4. OPs Ameliorate Gut Microbiota Dysbiosis in UC Mice

**Figure 4:**
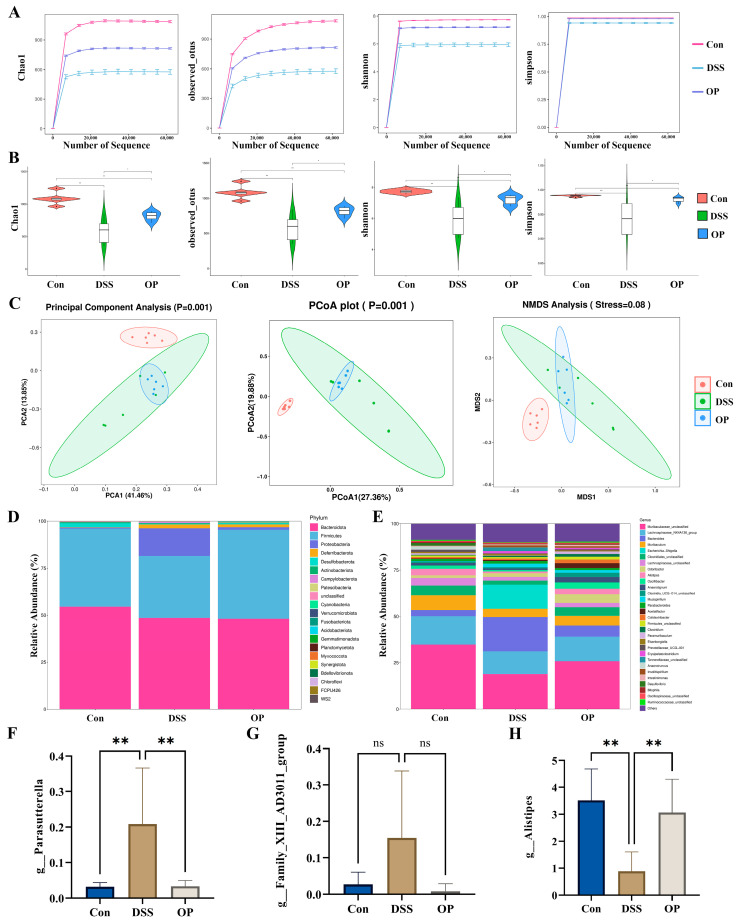
Effect of OP supplementation on the gut microbiota in UC mice. (**A**) Dilution curves for the Chao1, Observed OTU, Shannon, and Simpson indices, *n* = 6. (**B**) Results of α-diversity analysis for the Chao1, Observed OTU, Shannon, and Simpson indices, *n* = 6. (**C**) β-diversity as assessed by PCA, PCoA, and NMDS results, *n* = 6. (**D**) Microbial composition of the three groups of mice at the phylum level, *n* = 6. (**E**) Microbial composition of the three groups of mice at the genus level, *n* = 6. (**F**–**H**) Expression changes in Parasutterella, Family_XIII_AD3011_group, and Alistipes, *n* = 6. ns, *p* > 0.05. *, *p* < 0.05. **, *p* < 0.01.

As previously noted, DSS therapy changed the composition and structure of the gut microbiota in colitis-affected animals, indicating that alteration of the gut microbiota plays a significant role in the development of colitis in mice [34]. Thus, we postulated that by enhancing the gut microbiota, OPs may lessen the colitis caused by DSS in mice. Using high-throughput sequencing of the 16S rDNA gene, we looked into how OPs affected the intestinal microbiota of mice that had colitis brought on by DSS. The dilution curves of the Chao1, Observed OTU, Shannon, and Simpson indices in the sequencing results all reached a plateau, indicating that the sequencing results were credible (Figure 4A). When compared to the DSS group, the microbiota’s richness and diversity were considerably higher in the NC and OP groups, according to the Alpha diversity results (Figure 4B). The groups’ similarities and differences were evaluated using PCA, PCoA, and NMDS. The addition of OP therapy resulted in a reduction in the considerable separation between the DSS and NC groups (Figure 4C).

Following the DSS treatment, the abundance of Proteobacteria greatly rose and the abundance of Firmicutes significantly decreased, according to the results of the bar stacking plots, while the OP treatment upregulated the proportion of Firmicutes and downregulated the proportion of Proteobacteria (Figure 4D). In addition, we simultaneously explored changes in microbiota expression at the genus level and found that the expression of Prevotella_9 and Family_XIII_AD3011_group was upregulated in the DSS group and that the expression of Alistipes was significantly downregulated in the DSS group (Figure 4E–H). In conclusion, by reducing the expression of pathogenic bacteria and boosting the expression of helpful bacteria, the OP therapy enhanced the diversity and richness of the intestinal microbiota in DSS-induced colitis mice.

### 3.5. Correlation Analysis between UC and Gut Microbiota

**Figure 5:**
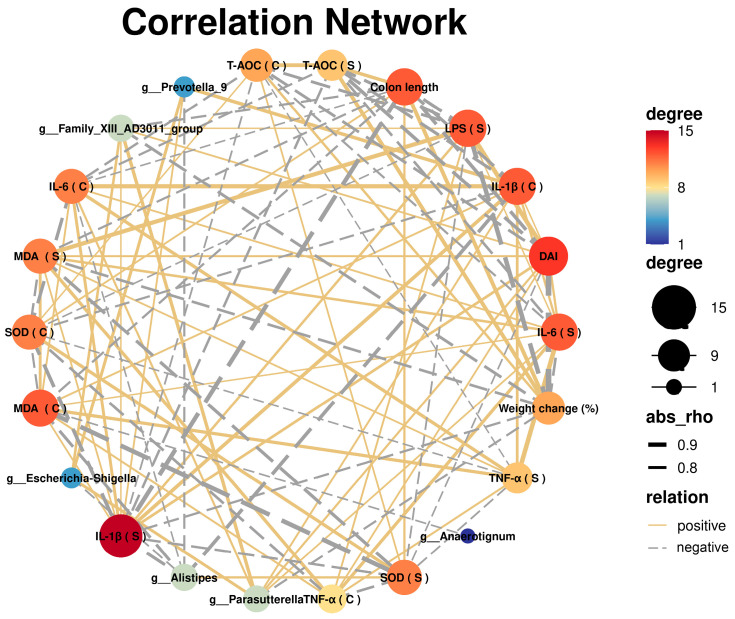
Network diagram of correlation analysis between gut microbiota and UC indicators: a correlation network was constructed using the OmicStudio tools at https://www.omicstudio.cn/tool on 16 October 2023.

Through the use of Spearman’s correlation analysis, it was possible to determine whether the OP treatment could potentially mitigate the symptoms and other parameters of DSS-induced colitis in mice by altering the relative abundance of gut microbiota that was affected by the OP treatment (Figure 5). The severity of colitis in mice was favorably connected with the number of Escherichia-Shigella, Prevotella_9, Parasutterella, and Family_XIII_AD3011_group, and negatively correlated with the abundance of Anaerotignum and Alistipes.

### 3.6. OPs Alleviated Colonic Inflammation by Inhibiting the TLR4/NF-κB Signaling Pathway

**Figure 6:**
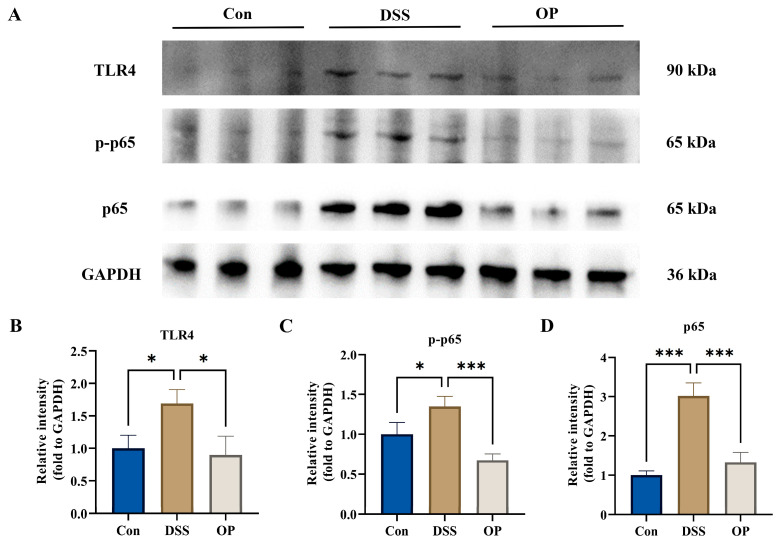
Effects of OP on the TLR4/NF-κB signaling pathway. (**A**) Differential expression of TLR4, p65, *p*-p65, and GAPDH in the three groups, *n* = 3. (**B**–**D**) Relative levels of TLR4, p65, and *p*-p65, *n* = 3. *, *p* < 0.05. ***, *p* < 0.0001.

The OPs were able to inhibit DSS-induced inflammatory responses in mice (Figure 3), and the NF-κB signaling pathway has been shown to exacerbate the worsening of colitis [35]. TLR4, p65, and *p*-p65 levels were found to be elevated in the DSS group in the current study when compared to the NC group (Figure 6A–D), indicating that the TLR4/NF-κB pathway was triggered. TLR4, p65, and *p*-p65 expression levels were all markedly lowered after the OP treatment, indicating that the OPs were able to prevent the TLR4/NF-κB pathway from being activated. All of these findings point to the possibility that OPs can reduce the inflammatory reactions that DSS causes in mice by blocking TLR4/NF-κB.

## 4. Discussion

Globally, the prevalence of UC is increasing year after year, and UC has become a global disease impacting public health [36]. Due to its incurable nature and severe recurrent effects, the quality of life of patients is severely affected [37]. Clinically, antibiotics, immunosuppressants, and glucocorticoids are widely used to alleviate UC; however, prolonged use of these drugs can lead to serious side effects and complications [38]. Marine-derived bioactive peptides have been reported to have high activity, low toxicity, and anti-inflammatory and antioxidant biological functions [39,40]. In the present study, we determined the mitigating effect of oyster peptides on colitis in mice. We found that OPs significantly alleviated the typical pathologic features of colitis in mice, such as weight loss, colon shortening, elevated disease scores, and increased inflammatory response. We also found that OPs restored the number of goblet cells, enhanced mucin expression, and improved the protein level of the tight junction protein claudin-1. Furthermore, feeding mice a meal supplemented with OPs improved the dysbiosis of gut microbiota and decreased the relative presence of the pathogenic bacterium Parasutterella. These findings imply that OP supplementation in the diet can successfully shield mice from DSS-induced colitis.

The intestinal barrier protects the body from invasion by foreign pathogenic microorganisms and reduces colonic injury, and the transmembrane barrier protein claudin-1 is an important component of the intestinal barrier [41,42]. The mucus layer, which is primarily made up of intestinal goblet cells and mucins, is the first physical barrier that bacteria face in the gut, in addition to tight junction proteins [43]. Impaired intestinal barrier function allows bacteria and their harmful substance LPS to enter the bloodstream, which triggers systemic inflammation and can exacerbate UC [44]. Proinflammatory cytokines have been reported to be one of the pathologic factors contributing to intestinal and mucosal inflammation [45]. The results of our study similarly showed that DSS induced inflammatory infiltration in mouse colonic tissues and significantly promoted the expression of proinflammatory cytokines in the colon and serum, whereas the OP treatment suppressed inflammation. Previous studies have shown that many natural marine products, such as oyster polysaccharides, sea cucumber peptides, and tuna bioactive peptides, are able to improve intestinal barrier function and inhibit the inflammatory response to improve colitis [19,24,46]. In this study, OP alleviated the impairment of the intestinal barrier caused by DSS.

Gut microbial disorders are important triggers in the pathogenesis of UC [47]. It has been shown that DSS induces gut microbial dysbiosis in mice, which in turn exacerbates the progression of UC [48]. Typically, Firmicutes and Bacteroidota dominate the gut microbiota, and an abnormal increase in the relative abundance of Proteobacteria c is a sign of an imbalance in gut microbiota [49]. Furthermore, a class of good bacteria called Firmicutes inhabits the gut, and disruption of the gut barrier lowers the relative abundance of Firmicutes [50,51]. In this study, the administration of OPs improved the dysbiosis of the gut microbiota, while the relative abundance of Firmicutes fell and Proteobacteria increased in the DSS group. The abundance of Firmicutes was positively correlated with mouse colon length and T-AOC and negatively correlated with inflammatory factors, DAI scores, and weight loss. Conversely, Proteobacteria was positively correlated with the severity of colitis in mice. Notably, the relative levels of Alistipes were significantly higher in the OP group than in the DSS group. Furthermore, there was a negative correlation between the abundance of Alistipes and inflammatory markers, DAI score, and weight loss, and a positive correlation with colon length and T-AOC. Alistipes has been shown to be not only a beneficial gut microorganism but also a short-chain fatty acid (SCFA)-producing bacterium [52,53]. In addition, many studies have shown that Parasutterella is highly expressed in UC mice and is a potentially harmful class of bacteria, which is consistent with our findings [54,55]. In this study, there were positive and negative correlations found between Parasutterella and colon length and T-AOC, as well as positive correlations with inflammatory markers, DAI score, and weight loss. When the OPs were applied, the relative abundance of Parasutterella was considerably lower than in the DSS group. The aforementioned findings imply that OPs may alleviate colitis by correcting the gut flora’s imbalance.

Imbalances in gut microbes usually lead to an increase in harmful bacteria and a decrease in beneficial bacteria [31]. The TLR4/NF-κB signaling pathway plays a critical role in the progression of colitis in mice and can mediate biological processes such as immunity and inflammation [56]. LPS produced by harmful bacteria can bind TLR4 on the cell membrane surface and promote the expression of inflammatory factors by activating the NF-κB signaling pathway [57]. The results of this study showed that the DSS treatment activated the TLR4-NFκB signaling pathway in the mouse colon, while the OP treatment significantly inhibited the pathway. The above results suggest that OPs are able to ameliorate DSS-induced colitis in mice by inhibiting the TLR4/NF-κB signaling pathway and thereby ameliorating DSS-induced colitis.

Bioactive peptides are specific amino acid fragments of proteins that not only have nutritional value but also have beneficial effects on health [58]. For example, wheat peptides were able to alleviate DSS-induced colitis in mice by activating the NRF2-Keap1 signaling pathway and thereby alleviating DSS-induced colitis [30]. Atrial natriuretic peptide was able to attenuate colitis in mice by inhibiting the cGAS-STING pathway [59]. Related studies have focused on exploring the biological activity of individual peptides. For example, the Trichinella matsutake-derived peptide WFNNAGP prevents DSS-induced colitis by improving oxidative stress and intestinal barrier function [60]. The walnut-derived peptide LPF alleviated colitis by reducing apoptosis, reducing inflammation, and modulating the gut microbiota [61]. Numerous bioactive peptides generated from marine food have been shown to exhibit a range of biological properties, including anti-inflammatory, antioxidant, and anti-obesity properties [62]. In the present study, oyster peptides were similarly able to alleviate the symptoms of DSS-induced colitis by maintaining intestinal barrier integrity, modulating the gut microbiota, and inhibiting the TLR4/NF-κB signaling pathway.

## 5. Conclusions

In this study, we demonstrated that in colitis-affected animals, OPs enhanced the quantity of goblet cells and upregulated the production of mucin and the tight junction protein claudin-1. Additionally, OPs lessen the relative abundance of Proteobacteria, lower the intestinal microbial imbalance brought on by DSS, and lessen the oxidative damage and inflammation in the gut. Furthermore, the OP therapy reduced inflammatory reactions brought on by DSS by blocking the TLR4/NF-κB signaling pathway. In addition, we characterized the peptide composition of the oyster peptides, and in the following studies, we will explore the potential bioactive peptides in the oyster peptides. In summary, the OPs effectively alleviated DSS-induced colitis by improving the intestinal barrier and intestinal microbiota, and the results of this study provide innovative perspectives for the development of marine-food-derived bioactive peptides as functional foods to maintain intestinal health.

## Data Availability

The original contributions presented in the study are included in the article, further inquiries can be directed to the corresponding authors.

## Supplementary Materials

The following supporting information can be downloaded at: https://www.mdpi.com/article/10.3390/nu16111591/s1, Table S1: All primers; Table S2: Amino acid composition of OP; Table S3: Peptide identification of OP.
